# Short and Long term glycemic control among Type 2 DM patients in a resource-limited setting

**DOI:** 10.4314/ahs.v25i2.23

**Published:** 2025-06

**Authors:** Ijeoma Angela Meka, Chika Juliet Okwor, Ekene Joy Arum, Ochuko Otokunefor, Obumneme Benneth Anyim, Michael Ikechukwu Ogamba

**Affiliations:** 1 Department of Chemical Pathology, College of Medicine, University of Nigeria/University of Nigeria Teaching Hospital, Ituku/Ozalla, Enugu State, Nigeria; 2 Department of Chemical Pathology, College of Health Sciences, University of Port Harcourt, Rivers State, Nigeria; 3 Department of Internal Medicine, University of Nigeria Teaching Hospital. Ituku/Ozalla, Enugu, Nigeria; 4 Department of Chemical Pathology, PAMO University of Medical Sciences, Port Harcourt, Rivers State, Nigeria

**Keywords:** HbA1c, Fasting Plasma glucose, Diabetes Mellitus, glycemic control, discordance

## Abstract

**Background:**

Diabetes Mellitus is a chronic disease condition and one of public health importance in Africa and indeed globally. Its potential complications can be mitigated by tight control of blood glucose, achievable by both short and long term glucose monitoring. The values of these measures are expected to both be within target, but for some reasons, sometimes these values become discordant.

**Objective:**

This study is aimed at determining the pattern of short and long term glycemic control prevalent among Type 2 diabetic patients in the study environment and the extent of the discordance between them.

**Methods:**

A cross-sectional study carried out at University of Nigeria Teaching Hospital, Enugu. Research participants comprised consenting adults with Type 2 Diabetes Mellitus. Fasting plasma glucose and glycated hemoglobin were used to assess short and long term glycemic control respectively.

**Results:**

The study included 148 participants (60 males and 88 females). Glycated haemoglobin (HbAlc) correlated significantly with Fasting Plasma Glucose (FPG) (P < 0.00001). Prevalence of optimal long and short term glycemic control was 42.6% and 35.8% respectively. The proportion of individuals with concordance between FPG and HbA1c was 116 (78.4%) while 32 (21.6%) had discordant values.

**Conclusion:**

Glycemic control, both long and short terms, was sub-optimal among participants. Discordance observed between HbA1c and FPG creates some dilemma in clinical decision making, and calls for guidelines and uniformity in the clinical management of these conditions.

## Introduction

Non-communicable diseases account for a great number of morbidity and mortality globally, and constitute major health and economic burden to individuals, societies and governments. In Africa, Diabetes Mellitus (DM) is of a major concern as an estimated 24 million adults were living with the disease in the African region in the year 2021 according to a report by the International Diabetes Federation^1^. World Health Organization (WHO) equally reported a rising global prevalence which is more in low- and middle-income countries. This is attributed to some factors including an increasing overweight and obesity prevalence, coupled with widespread physical inactivity^2^. Other factors include rapid urbanization with associated harmful diet and lifestyle changes^3^.

It went further to project that diabetes would be the 7^th^ leading cause of death by 2030^4^. Blacks are said to have a higher risk of developing diabetes than whites, and also more likely to develop complications. These are attributed to factors like genetic predisposition, obesity, insulin resistance, and poor glycemic control^5^,^6^,^7^. In Nigeria, DM prevalence has been progressively increasing. Over the period from 1989 to 1998, its prevalence was put at 0.8 – 1.43%^8^,^9^. However, in 2018, a systematic review^10^ documented a pooled prevalence of 5.77%, while a prevalence of 10.0% was recently documented in 2022^11^.

DM can be associated with varied complications including microvascular and macrovascular complications. These complications which can be acute or long term can be prevented by tight glycemic control amongst other risk-reduction strategies. This was strongly affirmed by several prospective randomized controlled trials which showed that good glycemic control was associated with significantly reduced rates of microvascular and neuropathic complications^12^,^13^,^14^.

Blood glucose control monitoring is commonly done either by measuring Fasting Plasma Glucose (FPG) for short term (daily monitoring) or glycated haemoglobin (HbA1C) for long term (over a period of 8 – 12 weeks) monitoring. This followed the development in 2010 when the American Diabetes Association adopted the International Expert Committee recommendation on the use of glycated hemoglobin with a threshold of ≥6.5% for diagnosis of DM and <7.0% as a reasonable HbA1C goal for non-pregnant adults^15^. Hence the higher the HbA1c value, the poorer the long term control.

DM places appreciable but varying burdens on individuals, families, societies and government. It is usually accompanied with increased direct medical costs, can significantly affect quality of life and also create social and emotional burdens on individuals and families. The treatment and care of diabetic patients consume considerable healthcare resources and equally results in some lost productivity by affected individuals, hence also places some burden on society and government. It is therefore essential that complications are prevented by available glycemic control monitoring measures, which in this regards are Fasting Plasma Glucose for the short term monitoring and glycated hemoglobin for the long term monitoring. For good glycemic control, it is expected that both values remain within optimal values/targets. However, sometimes a discordance is observed between FPG and HbA1c, whereby a patient may exhibit good FPG value and a deranged HbA1c value, or vice versa. This scenario often causes a diagnostic and/or clinical management dilemma as there are currently no international guidelines for the management of these cases. Some causes of high HbA1c and normal FPG include HbA1c measurement errors, anaemia associated with reduced red cell turnover like in iron deficiency anaemia, asplenia, hypertriglyceridemia, hyperbilirubinaemia, and tight glucose control for only a short period before meeting the doctor. While some causes of normal HbA1c and high FPG include acute illness, late night meal and poor medication compliance close to clinic visit and glucose testing^16^. Though a number of studies^17^,^18^,^19^ have assessed the (overall) correlation between FPG and HbA1c and reported strong positive results, there is still a dearth of studies assessing the discordance between the two parameters in individual patients which arise in daily clinical practice. This discordance can have significant implications in the diagnosis and clinical management of affected patients.

On this basis, the authors aimed to determine the pattern of short and long term glycemic control prevalent among Type 2 diabetic patients in the study environment and the extent of the discordance between them.

## Methods

### Study design

This is a cross-sectional hospital-based empirical study

### Study location

The study was carried out in University of Nigeria Teaching Hospital (UNTH), Enugu. UNTH is a government-owned tertiary healthcare institution located in the Southeast geopolitical zone of Nigeria. Geopolitical zones in Nigeria are administrative divisions constituting a number of states grouped together based on geographical location, similar ethnicity, economic activities and common political ideology^20^. Other attributes of UNTH are as described in a previous publication^21^.

### Study population

These comprised adults with Type 2 Diabetes Mellitus. Consenting participants aged 18 years and above and of both sexes were consecutively recruited from the Endocrinology Out-patient Clinic of UNTH.

#### Inclusion criteria

Adults with Type 2 Diabetes Mellitus

#### Exclusion criteria

Individuals attending the clinic for the first time, and not yet on antidiabetic medication, individuals who declined consent, pregnant women, individuals with hemoglobinopathies, Vitamin B12, iron and folate deficiency, chronic renal and liver failure, those requiring emergency medical attention, and those with acute diabetic complications.

### Sample collection and analysis

Five mls of venous blood samples were collected aseptically from participants after 10 -14 hours of fasting. This was aliquoted thus; 3 mls into fluoride oxalate bottles for FPG assay, and 2 mls into EDTA bottles for HbA1c assay. Though HbA1c does not require fasting but this was done to minimize the discomfort of having to collect blood samples twice from each patient. FPG was analyzed spectrophotometrically using the glucose-oxidase method^22^ (Randox, UK) while HbA1c was determined in whole blood using the enzymatic assay method^23^.

### Ethical considerations

Ethical clearance was obtained from College of Medicine Research Ethics Committee. Study-specific verbal informed consent was obtained from respondents after the study objectives were explained to them and confidentiality of data assured.

### Data Collection

Data were collected using a researcher-designed semi-quantitative researcher-administered questionnaire. Questionnaires were earlier pretested with ten patients who were not eventually included in the study. Feedback from the pretest was used to draft the final version of the questionnaire used for the study. Collected data were entered into a password-protected personal computer. Data were anonymized as study numbers were assigned to participants and used throughout the study. These measures were taken to ensure data confidentiality.

### Data analysis

Collected data were entered into a Microsoft Excel spreadsheet, and double-checked for accuracy. Data analysis was done using Stata version 13 (Stata Corp., USA). Pearson's correlation was used to determine the strength of correlation between HbA1c and FPG. Chi-square test statistic was used to analyze the associations between participants' duration of illness, gender and age to discordance between HbA1c and FPG. Point biserial correlation was used to determine correlation between presence of discordance and age. A P-value of less than 0.05 was considered as statistically significant.

### Sample size

Using Fisher's formular for sample size calculation and a DM prevalence of 10.0%,^11^ the calculated sample size gave 138.3. To compensate for attrition, an extra 5% was added giving an adjusted sample size of 145. A minimum of 145 participants as calculated was considered representative of the population size.

### Definition of terms

HbA1C goal for non-pregnant adults was put at <7.0%,15 while that of fasting plasma glucose was < 7.0 mmol/L.24

## Results

**Figure 1:**
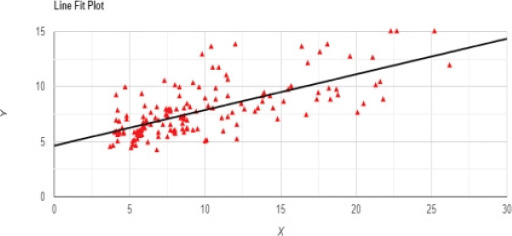
Scatter plot of FPG (X-axis) against HbA1c (Y-axis), r = 0.69, P < 0.00001

The study included 148 participants, comprising 60 males and 88 females with a Male: Female ratio of 1:1.5. The mean (SD) age of participants was 61.4 (11.2) years with range 26 to 86 years. Other demographic characteristics are as stated in Table 1. The participants' duration of the disease condition from time of diagnosis is as described in Table 2. There was a large positive significant correlation between HbA1c and FPG, r = 0.69, P < 0.00001 as shown in Figure 1.

**Table 1:** Demographic characteristics of participants

S/N	Demographics	Frequency (%)
1	Sex	
	Male	60 (40.5)
	Female	88 (59.5)
2	Age (years)	
	20 – 34	3 (2.0)
	35 – 49	13 (8.8)
	50 – 64	70 (47.3)
	65 – 79	52 (35.1)
	≥ 80	10 (6.8)
3	Marital Status	
	Single	5 (3.4)
	Married	135 (91.2)
	Widowed	8 (5.4)

**Table 2:** Duration of illness from time of diagnosis

S/N	Duration of illness from time of diagnosis	Frequency (%)
**1**	Less than 1 year	22 (14.9)
**2**	1 to < 5 years	37 (25.0)
**3**	5 years and above	89 (6**0**.1)

	**Total**	**148 (100.0)**

### Long term glycemic control

The mean (SD) HbA1c value was 7.8% (2.4).

The proportion of participants ‘Not at goal’ for HbA1c was 85 (57.4%). Hence the prevalence of optimal long term glycemic control was 63 (42.6%)

### Short term glycemic control

The mean (SD) for fasting plasma glucose was 9.8 (5.1) mmol/L.

The proportion of participants with deranged FPG values was 95 (64.2%), giving a prevalence of 53 (35.8%) for good short term glycemic control.

**Figure 2:**
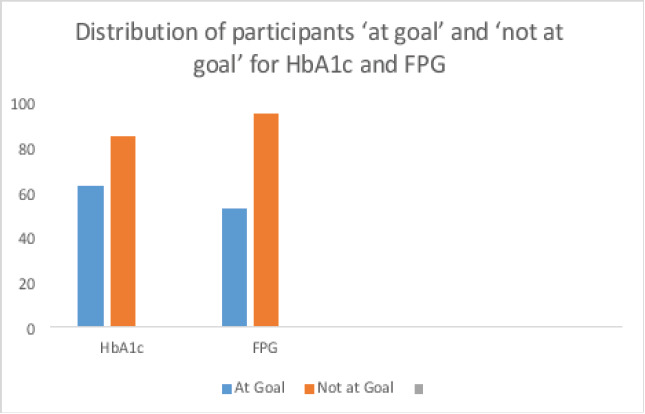
Distribution of participants ‘at goal’ and ‘not at goal’ for HbA1c and FPG

A greater proportion of participants was at goal for HbA1c (42.6%), compared to FPG (35.8%), though this was not statistically significant, (P = 0.2338), Figure 2.

### Discordance between FPG and HbA1c values among individual patients

The proportion of individuals with concordance between FPG and HbA1c was 116 (78.4%) while 32 (21.6%) had discordant FPG and HbA1c values. There was no statistically significant association between duration of illness, gender or age, to presence of discordance between FPG and HbA1c, Table 3. Presence of discordance was also not significantly correlated with age, r = 0.00605, P-value = 0.94187.

**Table 3:** Association of duration of illness, gender and age to discordance between HbA1c and FPG

S/N		Frequency (%) of discordance	Frequency (%) of no discordance	P-value
	Duration of illness from time of diagnosis			
**1**	Less than 1 year	6 (18.8)	16 (13.8)	0.0912
**2**	1 to <5 years	12 (37.5)	25 (21.6)	
**3**	5 years and above	14 (43.7)	75 (64.6)	
	Total	32 (100.0)	116 (100.0)	

	Gender			
**1**	Female	17 (53.1)	71 (61.2)	0.4097
**2**	Male	15 (46.9)	45 (38.8)	
	Total	32 (100.0)	116 (100.0)	

	Age			
**1**	20 – 34	1 (3.0)	2 (1.7)	0.9436#
**2**	35 – 49	2 (6.3)	11 (9.5)	
**3**	50 – 64	15 (46.9)	55 (47.4)	
**4**	65 – 79	12 (37.5)	40 (34.5)	
**5**	≥ 80	2 (6.3)	8 (6.9)	
	Total	32 (100.0)	116 (100.0	

# Fishers exact

### Participants' glycemic status according to gender, age and duration of illness

More than 50% of both males and females were not at goal for HbA1c and FPG. However, of those at goal for long and short term glycemic control, the proportion of females was higher than that of males, though this was not statistically significant, Table 4.

**Table 4:** Association of participants' glycemic status to gender

S/N	Gender	At goal (Freq, %)	Not at goal (Freq, %)	P-value
	**HbAlc**			
**1**	Male	28 (44.4)	32 (37.6)	0.405
**2**	Female	35 (55.6)	53 (62.4)	
		
	Total	63 (100.0)	85 (100.0)	
		
	**FBG**			
**1**	Male	23 (43.4)	37 (38.9)	0.5971
**2**	Female	30 (56.6)	58 (61.1)	
	
	Total	53 (100.0)	95 (100.0)	

Participants in the age range 65 - 79 years recorded the highest proportion of good long and short term glycemic control when compared with other age ranges, though this was also not statistically significant, Table 5.

**Table 5:** Association of participants' glycemic status to age

S/N	Age (years)	At goal (Freq, %)	Not at goal	P-value
	HbA1c			
**1**	20 - 34	2 (3.2)	1 (1.2)	0.0719#
**2**	35 - 49	4 (6.3)	9 (10.6)	
**3**	50 - 64	23 (36.5)	47 (55.3)	
**4**	65 - 79	28 (44.4)	24 (28.2)	
**5**	≥ 80	6 (9.5)	4 (4.7)	
	Total	63 (100.0)	85 (100.0)	

	**FPG**			
**1**	20 - 34	1 (1.9)	2 (2.1)	0.2371#
**2**	35 - 49	4 (7.5)	9 (9.5)	
**3**	50 - 64	20 (37.7)	50 (52.6)	
**4**	65 - 79	22 (41.6)	30 (31.6)	
**5**	≥ 80	6 (11.3)	4 (4.2)	
	
	Total	53 (100.0)	95 (100.0)	

# Fishers exact

In terms of duration of illness, those with illness duration 5 years and above had the highest proportion of long and short term glycaemic control. This finding was significant for FPG but not for HbA1c, Table 6.

**Table 6:** Association of participants' glycemic status to duration of illness

S/N	Duration of illness from time of diagnosis	At goal (Freq, %)	Not at goal	P-value
	**HbA1c**			
**1**	Less than 1 year	12 (19.0)	10 (11.8)	0.1319
**2**	1 year to < 5 years	19 (30.2)	18 (21.2)	
**2**	5 years and above	32 (50.8)	57 (67.0)	
	Total	63 (100.0)	85 (100.0)	

	**FPG**			
**1**	Less than1 year	12 (22.6)	10 (10.5)	0.0377
**2**	1 year to < 5 years	16 (30.2)	21 (22.1)	
**2**	5 years and above	25 (47.2)	64 (67.4)	
	
	Total	53 (100.0)	95 (100.0)	

## Discussion

The importance of long and short term monitoring of blood glucose in diabetics cannot be overemphasized. Glycated haemoglobin since its introduction has been received with great enthusiasm among diabetes care clinicians, as it has proven to be of great value in the care of diabetes patients and used as a marker for predicting long-term complications^25^. This study evaluates the long and short term control patterns as well as the discordance which exists between HbA1c and FPG. The positive correlation between HbA1c and FPG recorded in this study corroborates with a number of studies^17^,^18^,^19^. This supports the fact that glycosylation occurs more at higher blood glucose levels^26^.

The mean (SD) age of participants 61.4 (11.2) recorded in this study is similar to 62.2 (11.7) reported in a previous study^27^ carried out in Enugu. Majority of participants in the index study were in the age group 50 and above. This is in line with the established fact that Type 2 DM is considered Adult-Onset disease, and the peak age at diagnosis is in the fifth decade of life^28^,^29^.

The mean level for FPG reported in this study is similar to 10.62 (3.62) reported in Zaria, Nigeria^30^ and 8.6 (4.3) reported by Adebisi et al,^31^ but differs from 7.83 (2.22) recorded in Kathmandu^32^. On the other hand, the mean (SD) of 7.8 (2.4) % for HbA1c in the index study is similar to the mean of 8.0 reported by Adebisi et al^31^ and 8.6 (1.9) % reported in Finland^33^.

The proportion of participants who were ‘Not at goal’ for HbA1c as reported in this study is higher than 36.6% reported in Zaria, Nigeria^29^ as having suboptimal glycaemic control using HbA1c. It however comes close to the 54% reported in Lagos, Nigeria^34^ but is lower than 67% reported in Saudi Arabia^35^.

The proportion of participants with good long term control in this study varies with 58% reported in Benin, Nigeria^36^. In terms of short term control, the proportion of participants with poor control recorded in this study is higher than the 52.9% reported in a previous study^27^ but lower than the 76.7% reported in the Kathmandu study^32^. The differences observed between the present study and previous studies could be attributed to genetic and environmental differences in the populations studied, and also differences in the time of study.

The above findings (means and proportions of participants not at goal for HBA1c and FPG) unfortunately suggest poor long and short term glycemic controls among participants. Suboptimal glycemic control has some untoward consequences which include the risk of organ damage and progression of other diabetic complications. Short term control is particularly essential for monitoring acute changes in blood glucose, and is routinely used in daily management of cases. It is particularly useful for monitoring patients who are at risk of acute diabetic complications. Diabetic complications inadvertently reduce the quality of life of patients, increase cost of treatment and mortality risk. This finding of poor control is critical in our participant group as the goal of diabetes care is to maintain good glycemic control both in the long and short terms, essential for minimizing and mitigating complications. It also calls to question the level of utilization of HbA1c and adherence to established recommendations among clinicians. The American Diabetic Association's recommendation^37^ for HbA1c testing frequency is once every six months for stable patients, and more frequently for unstable patients and patients undergoing change in therapy. It remains to be confirmed if this recommendation is adhered to among patients and clinicians in Nigeria. The relatively high cost of HbA1c testing in Nigeria may also limit the frequency of testing among patients, particularly due to the fact that most patients engage in out-of-pocket funding of healthcare services.

In this study, more females had better short and long term glycemic control than males. A number of previous studies have reported better glycemic control in women^36^,^38^. while some reported better control in men^33^,^39^. Determinants of glycemic control are many and varied, and include social support, adherence to prescribed medication, cultural beliefs and practices, cardiovascular risk profiles, physiological responses to therapeutic interventions, amongst other factors. Hence, sex differences in glycemic control may be a complex interplay of these factors.

Discordance between HbA1c and FPG was recorded in this study. FPG within reference with an elevated HbA1c might be as a result of tight glucose control shortly before seeing the doctor and testing for FPG, poor adherence to medication but becoming compliant close to clinic appointment and testing for FPG, or postprandial hyperglycaemia. While elevated FPG and normal HbA1c may be due to late/large night meals, acute illness or intake of certain drugs like interferon-alpha. To the best of the authors' knowledge, this discordance has not been largely reported in Nigeria, but was recorded by Ho-Pharm et al in Vietnam^40^ and also by Abdul Murad et al in Malaysia^41^. When discordance is due to an obvious cause, it is easy to manage but when no obvious explanation is seen, it constitutes a management challenge to clinicians. Three propositions have been put forward to explain this discordance. First is that discordance may be due to glycemic fluctuations not captured in a small number of glucose measurements. Second is measurement variability in either glucose or HbA1c, usually traceable to point-of-care measurements, or to limits on assay standardization. The third explanation suggests that discordances are partly real and are due to intra-individual physiological mechanisms apart from plasma glucose fluctuations^42^.

There is a growing body of evidence supporting the third explanation, hence some investigators have proposed the use of glycation ‘gaps’ to quantify the discrepancies between plasma glucose and HbA1c. As we intensify efforts towards prevention of complications of diabetes, it remains imperative that stakeholders should make concerted efforts towards a unified interpretation of discordances between plasma glucose and HbA1c.

## Conclusion

The findings in this study suggest suboptimal short and long term glycemic control among participants. Presence of discordant HbA1c and FPG values was also noted. The data reported in this study provides evidence for quality improvement projects aimed at improving glycemic control among diabetes patients, and equally calls for the development of guidelines in the clinical management of individuals with discordant values.

## Study limitations

There is documented evidence that certain risk factors exist for sustained poor glycemic control. The present study was unable to capture these risk factors. The study was also not able to capture the frequency of testing for HbA1c practiced by clinicians in the study area. These limitations therefore represent gaps for future research.
